# Iron and Zinc Nutrition in the Economically-Developed World: A Review

**DOI:** 10.3390/nu5083184

**Published:** 2013-08-13

**Authors:** Karen H. C. Lim, Lynn J. Riddell, Caryl A. Nowson, Alison O. Booth, Ewa A. Szymlek-Gay

**Affiliations:** Centre for Physical Activity and Nutrition Research, School of Exercise and Nutrition Sciences, Deakin University, Burwood, Victoria 3125, Australia; E-Mails: k.lim@deakin.edu.au (K.H.C.L); lynn.riddell@deakin.edu.au (L.J.R.); caryl.nowson@deakin.edu.au (C.A.N.); alison.booth@deakin.edu.au (A.O.B.)

**Keywords:** iron, zinc, minerals, nutrients, diet, food, nutritional status, dietary requirements, adults

## Abstract

This review compares iron and zinc food sources, dietary intakes, dietary recommendations, nutritional status, bioavailability and interactions, with a focus on adults in economically-developed countries. The main sources of iron and zinc are cereals and meat, with fortificant iron and zinc potentially making an important contribution. Current fortification practices are concerning as there is little regulation or monitoring of intakes. In the countries included in this review, the proportion of individuals with iron intakes below recommendations was similar to the proportion of individuals with suboptimal iron status. Due to a lack of population zinc status information, similar comparisons cannot be made for zinc intakes and status. Significant data indicate that inhibitors of iron absorption include phytate, polyphenols, soy protein and calcium, and enhancers include animal tissue and ascorbic acid. It appears that of these, only phytate and soy protein also inhibit zinc absorption. Most data are derived from single-meal studies, which tend to amplify impacts on iron absorption in contrast to studies that utilize a realistic food matrix. These interactions need to be substantiated by studies that account for whole diets, however in the interim, it may be prudent for those at risk of iron deficiency to maximize absorption by reducing consumption of inhibitors and including enhancers at mealtimes.

## 1. Introduction

Iron and zinc are the two most abundant trace minerals in the human body, with 3–4 g of iron and 1.5–2.5 g of zinc present in the average adult [1,2]. In human nutrition, zinc and iron are frequently assessed together [3,4,5,6,7,8,9] as these minerals share common dietary sources, the absorption of both nutrients from food is believed to be enhanced and inhibited by similar compounds [4,5,7,9], and consequently, deficiency of both nutrients is thought to occur simultaneously. This review aims to discuss iron and zinc in terms of dietary sources, bioavailability and dietary interactions drawing on literature from human studies. In addition, we have reviewed dietary recommendations for iron and zinc, current intakes and nutritional status in older adolescents and adults in English-speaking, economically-developed countries. This paper communicates the most recent information regarding iron and zinc recommendations, nutrition surveys and nutrition research.

## 2. Methods

Two electronic databases, PubMed and EbscoHost, were used to undertake literature searches for studies examining iron and zinc bioavailability, and interactions between iron and zinc. The searches were conducted between February 2012 and May 2013. The terms used to identify relevant studies are shown in Table 1 below.

**Table 1:** Summary of literature search strategy.

	Search terms for iron and zinc bioavailability	Search terms for interactions between dietary iron and zinc
1	iron	iron
2	zinc	zinc
3	absorption	interaction
4	bioavailability	supplementation
5	absorption and bioavailability	iron and zinc
6	iron and absorption and bioavailability	interaction and iron and zinc
7	zinc and absorption and bioavailability	supplementation and iron and zinc

The searches were subsequently limited to studies published in the English language and those conducted in humans. In addition, reference lists of the retrieved original and review articles were searched to identify any other relevant studies.

Government publications were identified to determine current dietary recommendations for iron and zinc. National survey publications were identified to determine current iron and zinc intakes and status. Both types of publications were limited to data on older adolescents and adults in English-speaking, economically-developed countries.

**Figure 1:**
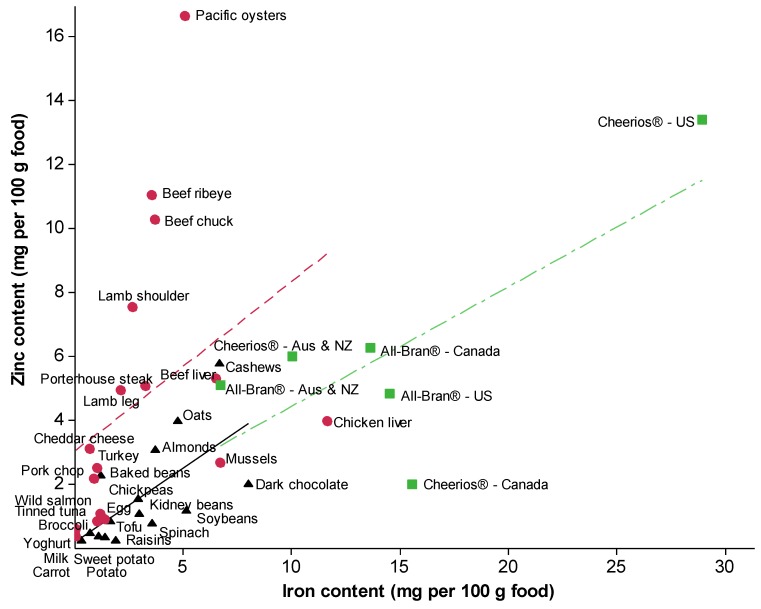
Relationship between dietary iron and zinc content of selected foods of plant origin (indicated by black triangles), foods of animal origin (indicated by red circles) (adapted from USDA National Nutrient Database [20]), and fortified breakfast cereals (indicated by green squares [15,16,17,18,19]). For foods of plant origin (black solid line): *r* = 0.68, *P* = 0.005. For foods of animal origin (red dash line): *r* = 0.71, *P* = 0.001. For fortified breakfast cereals (green dash-dot line): *r* = 0.87, *P* = 0.086. Significance does not change when the two outliers, Pacific oysters and US Cheerios^®^, are omitted. Values are based on uncooked nuts, carrot, oysters, raisins, oats and dairy products, and plain breakfast cereals; all other foods are cooked.

For Figure 1, Release 25 of the US Department of Agriculture National Nutrient Database for Standard Reference was used to source the iron and zinc content of foods identified in national surveys as contributing to iron or zinc intakes. Efforts were made to identify fortified breakfast cereals that are marketed across English-speaking countries. Nutritional information published online by manufacturers was found for All-Bran^®^ and Cheerios^®^ in Australia and New Zealand, the US, and Canada, but not the UK. The iron and zinc content of foods was plotted using Minitab 15 [10] and Spearman correlation tests were used to determine the associations between iron and zinc content according to whether foods are of plant origin, animal origin, or fortified with iron and zinc.

## 3. Dietary Sources and Intakes of Iron and Zinc

Foods recognized as good sources of iron tend to also contain zinc, the exception being dairy products which are a poor source of iron but recognized as a good source of zinc. Figure 1 depicts the association between iron content and zinc content in foods identified in national surveys as the main contributors of dietary iron or zinc intakes [11,12,13,14], as well as select fortified foods [15,16,17,18,19]. Using the foods in Figure 1 as an example, the association between iron and zinc content seems to be similar in foods of animal origin (*r* = 0.71, *P* = 0.001) and unfortified foods of plant origin (*r* = 0.68, *P* = 0.005).

The animal-derived foods presented in Figure 1 contain 189% more zinc than the unfortified plant-derived foods (mean 4.6 mg *vs.* 1.6 mg). Given this disparity, it is unsurprising that a recent meta-analysis found zinc intakes and zinc status to be lower among males and females following a vegetarian diet compared to those who consume meat [21]. The iron content of the foods in Figure 1 are more comparable, with animal-derived foods containing 0.64% less iron than unfortified plant-derived foods (mean 3.0 mg *vs.* 3.1 mg). Iron intakes have been found to be similar in those following a vegetarian or omnivorous diet [6,22,23,24,25], however the iron status of vegetarians is often lower [22,23,25,26]. It is widely accepted that iron and perhaps zinc in animal-derived foods are more bioavailable than the iron and zinc present in plant-derived foods, although a well-planned vegetarian diet can provide sufficient amounts of dietary iron and zinc [27].

Government agencies in the US, Canada, the UK, New Zealand and Australia have assessed population dietary iron and zinc intakes, and the US, the UK, New Zealand and Australia have also assessed the main dietary sources contributing to these intakes (Table 2, Table 3). As foods have been categorized differently in these national surveys (e.g., whether pasta or rice have been grouped with breakfast cereals and bread), foods have been grouped into broader categories in Table 2, Table 3 for consistency. Across the surveys and among males and females, the bulk of dietary iron and zinc was sourced from cereal and meat products. Vegetables contributed to iron but not zinc intakes, and dairy contributed to zinc but not iron intakes. Males had higher intakes of both iron and zinc than females. Females were more likely to have inadequate iron intakes than males in all countries. In contrast, due to the higher recommendations for males, males were more likely than females to have inadequate zinc intakes in all countries but the US (Table 2, Table 3).

The contribution of cereal foods to iron and zinc intakes is noteworthy as these foods are often targets for fortification. In economically-developed countries, such fortification tends to be market-driven (e.g., voluntary fortification of breakfast cereals) or targeted-fortification (e.g., infant formula), as opposed to mass-fortification (e.g., mandatory fortification of staple foods such as wheat flour) [28]. The balance of these three forms of iron and zinc fortification varies across countries. Mass-fortification of iron in wheat flour is mandatory in the US, Canada (each 44 ppm; began in the 1940s [29]) and the UK (16.5 ppm, began in 1953 [30]) but is not mandatory in Australia or New Zealand [31]. Mass zinc fortification is not mandatory in any English-speaking, economically-developed countries, and is only mandatory in countries which are not a focus of this review: Indonesia (30 ppm in wheat flour), Jordan (20 ppm in wheat flour), Mexico (16 ppm in wheat and maize flours) and South Africa (15 ppm in wheat and maize flours) [31,32]. The breakfast cereals displayed in Figure 1 are an example of the variation in market-driven fortification worldwide. These cereals demonstrate that the same product around the world can vary markedly in the concentrations of fortificant iron and zinc. Importantly, as displayed in Figure 1, there is a tendency for the levels of fortificant iron and zinc in the cereals to exceed the levels naturally present in most foods.

There is also variance in the regulation of voluntarily fortified foods across countries. In Australia and New Zealand, only certain foods are permitted to be vectors for fortificant iron and zinc, and the amount of fortificant is indirectly limited through labeling and marketing restrictions [33]. For example, manufacturers cannot claim more than 3.0 mg iron for every 50 g of bread [33], although there are no regulations preventing manufacturers from adding more than 3.0 mg per 50 g. Canada also has restrictions as to which foods may be fortified with iron and zinc, and in certain cases the Canadian regulations mandate the quantity that must be added to specific foods [34]. For instance, if iron or zinc are added to a breakfast cereal, the cereal must contain 13.3 mg of iron and/or 3.5 mg of zinc per 100 g so that iron- or zinc-fortified cereals all contain the same concentration [34]. While Canadian regulations stipulate upper limits on fortificant vitamin A, C, D, E and the B-group vitamins, there are no such limits for iron and zinc [34]. The European Union expressly permits the use of iron and zinc as fortificants [35], although the maximum level of fortification is at the discretion of individual Member States [36]. In the UK, market-driven fortification is not restricted by limits other than the food needing to be safe for human consumption [37,38]. The US also does not uphold limits of iron and zinc fortification. In the US, manufacturers are provided with guidelines for amounts of nutrients per 100 kcal based on the Recommended Dietary Allowance which they can choose to use [29]. The worldwide lack of constraint or limits on the amount of fortificant iron and zinc permitted in foods is concerning as accumulation of iron in the body can lead to heart and liver failure [39] and habitual, moderately-high intakes of zinc (*i.e.*, from 15 mg/day) may lead to impaired copper status, which may then adversely affect lipoprotein metabolism [40]. The contribution of fortified foods to nutrient intakes worldwide is largely unknown, with the US’ Institute of Medicine calling for government and industry groups to collaboratively investigate the impact [29]. Despite the lack of empirical evidence, the role of fortified foods must be considered as levels of fortification worldwide are unprecedented [41]. Accordingly, the proportional contributions of foods presented in Table 2, Table 3 should be interpreted cautiously as information from Australia, the UK, and the US is over 10 years old and may not reflect the current abundance of market-driven fortified foods. Importantly, with the exception of New Zealand, there is a lack of national data from a single survey period showing the intakes, adequacy of the intakes, and the main food sources of iron or zinc.

## 4. Recommended Intakes of Iron and Zinc

Many countries have quantified the dietary requirements of humans. These reference values are generally determined by a panel based on scientific evidence and are used to assess whether the intakes of individuals or groups are adequate [42]. For each nutrient, recommendations are derived from the frequency distribution of requirements of a group of individuals. The resulting values are an Estimated Average Requirement (EAR), which is generally the quantity meeting the needs of half the healthy individuals in a given life stage and sex group (*i.e.*, the 50th percentile) [43]. Another key reference value can be derived from the EAR, known as the Recommended Dietary Intake (RDI) in Australia and New Zealand, the Recommended Dietary Allowance (RDA) in the US and Canada, and the Reference Nutrient Intake (RNI) in the UK [42]. This value meets the requirements of nearly all individuals in a given life stage and sex group. Across the US, Canada, the UK, New Zealand and Australia, the EAR for iron ranges from 5 to 11.4 mg/day and the EAR for zinc ranges from 5.5 to 12 mg/day (Table 4). The European Union is currently reviewing dietary recommendations with intent to standardize recommendations across all Member States [44]. While recommendations vary across these economically-developed nations, there is consistency in that greater amounts of iron are recommended for women, and greater amounts of zinc are recommended for men.

**Table 2:** Intakes, adequacy, and top food sources of dietary iron in economically-developed countries.

Country	Survey	Sample	Dietary iron intake (mg/day)	Inadequate intakes (*i.e.*, intakes < EAR) %	Top 3 Food sources	Contribution of food to iron intake %
Australia	1995 National Nutrition Survey [11]	Males 19+ years (*n* = 5081)	Median: 15.2 ^a^		Cereals and cereal products (includes breads, breakfast cereals, biscuits, cakes)	41
					Meat and meat products (includes poultry)	22
					Vegetables	12
		Females 19+ years (*n* = 5770)	Median: 11.1 ^a^		Cereals and cereal products (includes breads, breakfast cereals, biscuits, cakes)	38
					Meat and meat products (includes poultry)	17
					Vegetables	13
Canada	2004 Community Health Survey [45]	Males 19+ years (*n* = 8470)	Median (25%–75% IQR): 15.7 (12.9–19.1)	0.4 (SE 0.1)		
		Females 19+ years (*n* = 10350)	Median (25%–75% IQR): 11.9 (9.9–14.3)	19–30 years: 17 (SE 1.5)		
				31–50 years: 18 (SE 1.1)		
				51–70 years: <3 ^a^		
				>70 years: 2 (SE 0.4)		
New Zealand	2008/2009 Adult Nutrition Survey [12]	Males 15+ years (*n* = 2066)	Median (25%–75% IQR): 13.2 (12.8–13.6)	1 ^a^	Cereals and cereal products (includes breads, breakfast cereals, biscuits, cakes)	40
					Meat and meat products (includes poultry)	20
					Vegetables	13
		Females 15+ years (*n* = 2655)	Median (25%–75% IQR): 9.9 (9.6–10.2)	10 ^a^	Cereals and cereal products (includes breads, breakfast cereals, biscuits, cakes)	36
					Vegetables	15
					Meat and meat products (includes poultry)	14
UK	Iron intake and inadequate intakes: 2008/09–2010/11 UK National Diet and Nutrition Survey [46] Food sources: 2000–2001 National Diet and Nutrition Survey [13]	Males 19–64 years (*n* = 346)	Median (lower 2.5 percentile-upper 2.5 percentile): 11.7 (5.7–22.3)	1 ^a,b^	Cereals and cereal products (includes breads, breakfast cereals, biscuits, cakes)	44
					Meat and meat products (includes poultry)	19
					Vegetables	9
		Females 19–64 years (*n* = 461)	Median (lower 2.5 percentile-upper 2.5 percentile): 9.7 (4.0–16.3)	22 ^a,b^	Cereals and cereal products (includes breads, breakfast cereals, biscuits, cakes)	45
					Meat and meat products (includes poultry)	15
					Vegetables	11
US	Iron intake: NHANES 2009–2010 [47]	Iron intake: Males 20+ years (*n* = 2789)	Mean (SE): 17.5 (0.43)	Males 19+ years <3 ^a^		
	Inadequate intakes: NHANES 2001–2002 [48]	Iron intake: Females 20+ years (*n* = 2973)	Mean (SE): 12.9 (0.33)	Females 19+ years 10 ^a^		
	Food sources: NHANES 2003–2006 [14]	Food sources: Males and females 19+ years (*n* = 9490)			Cereals and cereal products (includes breads, breakfast cereals, biscuits, cakes)	47
					Meat and meat products (includes poultry)	10
					Vegetables	6

^a^ No measure of spread or SE available; ^b^ Intakes below the Lower Reference Nutrient Intake (meets the requirements of 2.5% of the age and lifespan group). Abbreviations: NHANES—National Health and Nutrition Examination Survey; EAR—Estimated Average Requirement (meets the needs of 50% of individuals in the life stage group); IQR—interquartile range; SE—standard error.

**Table 3:** Intakes, adequacy, and top food sources of dietary zinc in economically-developed countries.

Country	Survey	Sample	Dietary zinc intake (mg/day)	Inadequate intakes (*i.e.*, intakes < EAR) %	Top 3 Food sources	Contribution of food to zinc intake %
Australia	1995 National Nutrition Survey [11]	Males 19+ years (*n* = 5081)	Median: 14.4 ^a^		Meat and meat products (includes poultry)	39
					Cereals and cereal products (includes breads, breakfast cereals, biscuits, cakes)	24
					Dairy products	12
		Females 19+ years (*n* = 5770)	Median: 9.7 ^a^		Meat and meat products (includes poultry)	32
					Cereals and cereal products (includes breads, breakfast cereals, biscuits, cakes)	24
					Dairy products	15
Canada	2004 Community Health Survey [45]	Males 19+ years (*n* = 8470)	Median (25%–75% IQR): 11.9 (10.3–13.8)	17 (SE 1.4)		
		Females 19+ years (*n* = 10350)	Median (25%–75% IQR): 9.5 (8.2–11.1)	14 (SE 1.3)		
New Zealand	2008/2009 Adult Nutrition Survey [12]	Males 15+ years (*n* = 2066)	Median (25%–75% IQR): 12.9 (12.4–13.4)	39 ^a^	Cereals and cereal products (includes breads, breakfast cereals, biscuits, cakes)	33
					Meat and meat products (includes poultry)	28
					Dairy products	11
		Females 15+ years (*n* = 2655)	Median (25%–75% IQR): 9.0 (8.7–9.3)	11 ^a^	Cereals and cereal products (includes breads, breakfast cereals, biscuits, cakes)	30
					Meat and meat products (includes poultry)	24
					Dairy products	14
UK	Zinc intake and inadequate intakes:2008/09–2010/11 UK National Diet and Nutrition Survey [46] Food sources:2000–2001 National Diet and Nutrition Survey [13]	Males 19–64 years (*n* = 346)	Median (lower 2.5 percentile-upper 2.5 percentile): 9.6 (4.7–20.1)	8 ^a,b^	Meat and meat products (includes poultry)	36
					Cereals and cereal products (includes breads, breakfast cereals, biscuits, cakes)	25
					Dairy products	16
		Females 19–64 years (*n* = 461)	Median (lower 2.5 percentile-upper 2.5 percentile): 7.6 (3.7–13.1)	3 ^a,b^	Meat and meat products (includes poultry)	30
					Cereals and cereal products (includes breads, breakfast cereals, biscuits, cakes)	26
					Dairy products	19
US	Zinc intake: NHANES 2009–2010 [47] Inadequate intakes: NHANES 2001–2002 [48] Food sources: NHANES 2003–2006 [14]	Zinc intake:Males 20+ years (*n* = 2789)	Mean (SE): 14.2 (0.22)	Males 19+ years 11 (SE 1.0)		
		Zinc intake:Females 20+ years (*n* = 2973)	Mean (SE): 9.8 (0.17)	Females 19+ years 17 (SE 1.6)		
		Food sources: Males and females 19+ years (*n* = 9490)			Meat and meat products (includes poultry)	35
					Cereals and cereal products (includes breads, breakfast cereals, biscuits, cakes)	15
					Dairy products	13

^a^ No measure of spread or SE available; ^b^ Intakes below the Lower Reference Nutrient Intake (meets the requirements of 2.5% of the age and life stage group). Abbreviations: NHANES—National Health and Nutrition Examination Survey; EAR—Estimated Average Requirement (meets the needs of 50% of individuals in the life stage group); IQR—interquartile range; SE—standard error.

**Table 4:** Recommended intakes of iron and zinc in Australia and New Zealand, UK, and US and Canada.

		Australia and New Zealand [49]		UK [50]		US and Canada [43]	
		EAR (mg/day)	RDI (mg/day)	EAR (mg/day)	RNI (mg/day)	EAR (mg/day)	RDA (mg/day)
**Iron**	Males 19–50 years	6	8	6.7	8.7	6	8
	Males 51+ years	6	8	6.7	8.7	6	8
	Females 19–50 years ^a^	8	18	11.4	14.8	8.1	18
	Females 51+ years	5	8	6.7	8.7	5	8
**Zinc**	Males 19–50 years	12	14	7.3	9.5	9.4	11
	Males 51+ years	12	14	7.3	9.5	9.4	11
	Females 19–50 years ^a^	6.5	8	5.5	7.0	6.8	8
	Females 51+ years	6.5	8	5.5	7.0	6.8	8

^a^ Non-pregnant and non-lactating women. Abbreviations: EAR—Estimated Average Requirement (meets the needs of 50% of individuals in the life stage group); RDI/RNI/RDA—Recommended Dietary Intake/Reference Nutrient Intake/Recommended Dietary Allowance (meets the needs of nearly all individuals in the life stage group).

## 5. Bioavailability

The chemical forms of iron and possibly zinc in food influence the amount absorbed through the gastrointestinal tract and into the bloodstream. In food, iron is present as heme iron and inorganic non-heme iron, with heme iron present exclusively in meats and non-heme iron present across the food chain in plants, eggs, milk, and meats [51]. It is understood that the human body regulates iron status through absorption rather than secretion [52], thus absorption of heme and non-heme iron has been widely studied, but much remains unknown [53]. Heme iron is readily absorbed through the small intestine, whereas non-heme iron is susceptible to being affected by compounds that impede or facilitate absorption and is less bioavailable [51]. It has been suggested that zinc is found as organic complexes with protein in meats, and as inorganic salts in plant foods [54,55], however no absorption studies (*i.e.*, using isotopic labeling) have been conducted in humans to determine whether these forms of zinc differ in bioavailability. In contrast to iron, the body regulates zinc homeostasis through gastrointestinal secretion and excretion of endogenous zinc in addition to absorption of exogenous zinc [43,56]. As zinc secretions are a major contributor to zinc status, it is possible interference with absorption has less of an impact on zinc status compared to iron, although research investigating this premise is required.

## 6. Inhibitors and Enhancers of Absorption

Iron and zinc are noted to interact with other meal components resulting in enhancement or inhibition of absorption through the intestine wall and into the bloodstream [57,58]. The absorption studies that have explored these interactions have generally tested absorption or fractional absorption from single meals using the widely-accepted stable isotopic or radioisotopic labeling techniques [56,59,60]. In these methods, stable isotopes or radioisotopes of iron or zinc are placed in a “carrier” meal (e.g., a bread roll or more complex food matrices including hamburger meals) that is consumed with or without a meal component suspected to influence absorption. To estimate iron absorption, incorporation of these stable isotopes or radioisotopes into erythrocytes or whole body counting of radioactivity is measured, while stable isotopes or radioisotopes in feces, urine, or the whole body are measured to estimate zinc absorption [61]. The amount of iron or zinc absorbed in the presence of the meal component in question is then compared to the amount absorbed without that meal component. In general, participants act as their own control, consuming the isotopes with and without the meal component of interest. The majority of absorption research has investigated iron, in particular non-heme iron, and the meal components investigated include phytate, polyphenols, animal tissue, soy protein, calcium, and ascorbic acid.

### 6.1. Phytate

Phytic acid (*myo*-inositol hexakisphosphate; IP_6_) and its salts (phytates) are the main storage form of phosphate in seeds and grains [62,63,64]. Chemically, phytic acid and phytate are known to be powerful chelators, binding to metals and forming insoluble compounds [63], and for this reason, it is thought phytate may impede absorption of nutrients such as iron and zinc. Single-meal studies of phytate in bran or as sodium phytate have demonstrated that as little as 2 mg phytate can reduce iron absorption by 18% [65]. These studies have also shown that the inhibitory effect increases with the phytate content, such that 250 mg phytate in a meal can reduce absorption by 82% [65,66]. Furthermore, hydrolyzing phytate by dehulling grains, adding phytase or hydrochloric acid, or catalyzing the action of endogenous phytase (e.g., soaking bran for 24 h at 55 °C) minimizes the inhibition of iron absorption [66,67,68], with catalysis of endogenous phytase increasing absorption by 119% [66]. There is also some evidence that other *myo*-inositol phosphates that contain fewer phosphate groups than IP_6_ (e.g., IP_3_, IP_4_, IP_5_) may also impede iron absorption [69]. The influence of phytate on zinc absorption has not been as extensively investigated. Larsson *et al.* [68] and Fredlund *et al.* [69] have indicated phytate may inhibit zinc absorption, although the study by Fredlund *et al.* pooled the absorption data of all participants rather than using a stronger cross-over design [70]. Thus, evidence for the inhibiting effect of phytate is weaker for zinc.

Absorption studies of phytate have tested doses of up to 250 mg phytate per meal. In a review of studies that have estimated phytate intakes around the world, Reddy [71] found omnivorous adult intakes to range from 395 to 1293 mg/day in the US and the UK [71]. As small amounts of phytate can reduce iron absorption significantly, it is likely that dietary phytate does contribute to reduced iron absorption in Western diets, however interactions throughout the whole food matrix may temper the chelating strength of phytate [72]. Although the findings of single-meal absorption studies may therefore not necessarily apply to a varied, whole diet, this premise has not been satisfactorily tested in a long-term dietary study. To investigate whether long-term consumption of phytate can affect iron status, Bach Kristensen *et al.* asked premenopausal women to consume high-fiber bread or dephytinized high-fiber bread for four months [73]. However, women receiving the dephytinized bread in this study still received a considerable amount of phytate (daily serve of 300 g dephytinized bread contained approximately 456 mg *vs.* 549 mg phytate in non-dephytinized bread), and accordingly, there was no difference in iron status [72]. A similar study design utilizing a more drastic reduction of phytate content may provide stronger evidence.

### 6.2. Polyphenols in Beverages

Polyphenols are a class of antioxidants, and complex combinations of polyphenols are found in foods and beverages sourced from plants including tea and coffee [74]. Single-meal studies using tea and coffee as sources of polyphenols have shown that polyphenols decrease iron absorption [75,76,77,78,79]. Although few studies have measured the polyphenol content of test beverages, it appears polyphenols have a dose-dependent effect on iron absorption, with increasingly concentrated tea having more potency [77]. Tea made from 3 g tea leaves concentrated at 396 mg/275 mL serve polyphenols reduced absorption by 91%, while the same tea diluted to 99 mg/275 mL serve polyphenols reduced iron absorption by 82% [77]. The timing of beverage consumption may also be influential. This notion was demonstrated by Morck *et al.* [78], who found that consuming coffee one hour following a meal or simultaneously with a meal both inhibited iron absorption by 44%, while coffee consumption one hour prior to food had no effect. The specific polyphenolic compounds responsible for the decreased iron absorption have not all been identified, although the influence of catechin polyphenols present in these beverages (e.g., epigallocatechin-3-gallate) has been demonstrated in Caco-2 cell models of human iron absorption [80]. While the inhibitory properties of polyphenols on iron absorption have consistently been demonstrated in absorption studies, complications arise when translating these findings into dietary recommendations. The quantity and composition of polyphenols varies widely, for example the total amount of catechins in tea made from 100 g of dry black tea may range from 1019 to 12,480 mg, and the processing of tea leaves, form (e.g., tea bag *versus* loose leaf) and brewing method (including amount and temperature of water and brew time) further affect the resulting content of polyphenols [81]. Adding milk to beverages does not appear to meaningfully alter the inhibitory effect of polyphenols [77].

No isotopic zinc absorption studies have been conducted for beverage polyphenols. Ganji and Kies [82] have used zinc balance methodology to measure the effect of tea. Participants resided in a metabolic ward and all input and output of zinc was measured with or without the co-consumption of tea [81]. Tea consumption did not affect zinc balance [82], however as the body uses endogenous intestinal secretions to regulate zinc metabolism, balance techniques do not provide an estimate of zinc absorption [56,61].

### 6.3. Animal Tissue

Findings from absorption studies suggest that different animal tissues have varied impacts on iron absorption [83,84]. Consuming animal tissue such as pork, lamb, beef, chicken or fish with meals seem to have comparable enhancing effects on iron absorption [83,84], while egg, casein and dairy foods have no such effect [84,85]. Furthermore, it appears that incorporating animal tissue in meals can counteract the inhibitory effect of phytate [86,87]. Consuming 196 g red kidney beans (227 mg phytate) with 100 g salmon has been shown to improve iron absorption by 48% [86]. The quantity of animal protein seems to be critical: 50 g of pork increased iron absorption from a rice meal (220 mg phytate) by 44%, but no effect was observed when just 25 g of pork was incorporated into the meal [87]. It is unknown whether the amount of protein required differs depending on the source animal. The inclusion of animal tissue, likely at least 50 g, to a meal is expected to improve iron absorption. However, a longer-term study comparing a self-selected five-day diet containing 222 g/day of meat, fish and poultry found no difference in iron absorption [88]. Therefore, the exact quantity and type of animal protein required remains unknown.

The influence of animal tissue on zinc absorption has been assessed by Sandström *et al.* [89], who observed increased zinc absorption when chicken was combined with a meal, while fish, milk, cheese and beef had no effect. However, this study was limited as it was not a cross-over study where participants acted as their own control. Rather, the results for all participants were pooled, thus the true effect on zinc absorption is not known.

### 6.4. Soy Protein

Up to 30 g of soy protein has been demonstrated to be an inhibitor of both iron and zinc absorption in single-meal studies [90,91,92,93]. While the phytate present in soy products contributes to the inhibition of iron absorption, some interference with absorption remains when phytate is removed, suggesting other compounds in soy are likely to be implicated [90]. The interactions between phytate, soy and zinc absorption are not yet understood [57].

### 6.5. Calcium

Results from studies investigating the influence of calcium from food and supplements on iron absorption have been conflicting. Studies assessing the effect of calcium naturally present in dairy foods have produced mixed results. In single-meal studies, consuming 150 mL milk (165 mg calcium) with bread rolls inhibited iron absorption by 57% [94], while 250 mL milk [76], 150 g milk and 125 g yoghurt [95] with more complex meals (e.g., steak meal, hamburger meal) had no effect. In relatively longer absorption studies measuring iron absorption over five days, consumption of dairy products inhibited absorption when all meals were provided [96] but no effect was observed when diets were self-selected by participants [97]. Single-meal and single-day studies indicate that supplemental or fortificant calcium has an inhibitory effect on heme and non-heme iron absorption [94,98,99,100], with as little as 40 mg fortificant calcium reducing absorption by 49% [93]. The inhibitory effect of supplemental calcium demonstrated in these short-term absorption studies has not translated to impaired iron status, with consumption of supplemental calcium (1000–1200 mg/day calcium) for 12 weeks to six months having no impact on iron status [98,101].

Studies assessing the effect of calcium on zinc absorption or zinc balance seem to indicate that fortificant and supplemental calcium have no effect [91,102,103,104].

### 6.6. Ascorbic Acid

Ascorbic acid has consistently been shown to enhance iron absorption when provided in single-meal studies as food sources [9,76,105,106] or in fortificant form [79,105]. Importantly, the improvement in iron absorption seems to vary depending on the composition of the meal, with 50 mg ascorbic acid (equivalent to approximately 100 g of fresh orange) increasing absorption by 61% from a hamburger meal and 164% from a pizza meal [105]. Similarly, iron absorption from bread rolls containing as much as 250 mg phytate was improved with the addition of 50 mg ascorbic acid [105], however 100 mg of ascorbic acid added to a meal of rice and soup could not counteract the inhibitory effect of tea [75]. The influence of meal composition and the whole food matrix may also explain why the dramatic improvements in iron absorption seen in single-meal studies were attenuated to a non-significant 35% improvement in a self-selected five-day diet study (247 mg/day ascorbic acid *vs.* 51 mg/day) [107]. The importance of considering the whole food matrix in relation to iron absorption, rather than individual food components, has further been demonstrated in long-term studies that have assessed whether consumption of ascorbic acid can affect iron status: while having 164 mg ascorbic acid in fruit with a breakfast cereal containing 272 mg phytate as a daily breakfast meal improved iron status [108], having 113 mg ascorbic acid was not sufficient to overcome the inhibitory effect of nearly 3000 mg phytate each day, and did not affect iron status [109]. Limited research suggests that other organic acids such as citric acid, malic acid and tartaric acid may also enhance iron absorption [110].

While evidence for the impact of ascorbic acid on iron absorption is strong, no studies have demonstrated any such effect on zinc absorption. Single-meal studies measuring zinc absorption with ascorbic acid naturally present in a food source [9] or added as a fortificant [111] found no enhancing or inhibiting effects.

## 7. Iron and Zinc Losses

Homeostatic regulation of iron and zinc differ, with iron being regulated through absorption and zinc being regulated primarily through secretion. As the body does not have a means to eliminate excess iron, absorption from the small intestine is tightly regulated by hepcidin. Hepcidin is a peptide hormone that is present in higher concentrations when body iron is replete [52]. Higher concentrations of hepcidin prevent ingested iron from entering the bloodstream by trapping iron in enterocytes which are naturally shed every two days [112], thereby preventing body iron from escalating to dangerous levels. In comparison, endogenous (pancreatic, biliary and intestinal) secretions comprise the main route of zinc loss, with larger zinc intakes being balanced by larger zinc secretions [113,114]. The main routes of iron and zinc losses are outlined in Table 5. The losses of iron in menstrual blood and zinc in ejaculate account for the sex-specific differences in dietary recommendations presented in Table 4.

**Table 5:** Main routes of iron and zinc losses.

Main routes of iron loss	Main routes of zinc loss
Urine (<1 mg/day)	Endogenous intestinal losses (0.5 to 3 mg/day)
Feces (from occult blood loss and shed enterocytes; 0.6 mg/day)	Urine (0.5 to 0.7 mg/day)
Menstrual blood (0.4 to 0.5 mg/day)	Skin cells (0.5 to 0.7 mg/day)
Skin cells and sweat (0.2 to 0.3 mg/day)	Semen (1 mg/ejaculate)

Adapted from 1, 2, 51, 113.

## 8. Iron and Zinc Status

### 8.1. Iron Status

Serum ferritin reflects the level of iron stores in the body [51] and is often used as a marker of iron status in population surveys. Table 6 displays the mean serum ferritin concentrations measured in recent population surveys of economically-developed nations and the prevalence of adequate or inadequate concentrations. Varied age classifications and cut-off values for adequacy prevent direct comparisons between countries, although it is evident that compared to men, women have lower serum ferritin and are more likely to have suboptimal serum ferritin concentrations. In women, serum ferritin concentrations seem to increase with age in line with menopausal status (Table 6). In considering Canada and New Zealand, the only two nations that have categorized both iron intakes (Table 2) and iron status of males and females (Table 6), women, particularly women of childbearing age, are less likely to meet the EAR for iron and are at greater risk of low iron stores. It is likely that the prevalence of low iron stores is higher than indicated in Table 6. Ferritin is a positive acute-phase response protein that increases during inflammation, thus in a state of inflammation, it no longer reflects the size of iron stores [51]. As only the New Zealand survey accounted for the effects of inflammation (*i.e.*, excluding participants with high levels of inflammation), low iron stores may be masked by inflammation in the remaining surveys.

**Table 6:** Measurements of ferritin and prevalence of low ferritin in national surveys.

Country	Survey	Sample	Ferritin (mean (95% CI) µg/L)	Low ferritin %
Canada	2009–2011 Canadian Health Measures Survey [115]	Males 20–49 years ^a^	164 (152–177) ^b^	1 ^c^
		Males 50–64 years ^a^	166 (148–187) ^b^	1 ^c^
		Males 65–79 years ^a^	155 (138–175) ^b^	0 ^c^
		Females 20–49 years ^d^	41 (38–44) ^b^	9 ^c^
		Females 50–64 years ^d^	85 (73–98) ^b^	6 ^c^
		Females 65–79 years ^d^	89 (74–107) ^b^	6 ^c^
New Zealand	2008/09 New Zealand Adult Nutrition Survey [12]	Males 15+ years (*n* = 1470)	177 (165–189) ^e^	2 ^f^
		Females 15+ years (*n* = 1878)	79 (75–84) ^e^	8 ^f^
UK	2008/09–2010/11 UK National Diet and Nutrition Survey [116]	Males 19–64 years (*n* = 160)	155 ^e,g^	
		Females 19–64 years (*n* = 231)	53 ^e,g^	
US	NHANES 2003–2006 [117]	Females 20–39 years ^h^ (*n* = 1780)	36.5 (33.5–39.9) ^b^	Females 20–49 years (*n* = 2539):13 ^c^
		Females 40–49 years ^h^ (*n* = 759)	40.0 (34.5–46.3) ^b^	

^a^ Total *n* = 1684 Canadian males aged 20–79 years; ^b^ Geometric mean ^c^ Low ferritin defined as <15 μg/L; ^d^ Total *n* = 1975 Canadian females aged 20–79 years; ^e^ Arithmetic mean ^f^ Low ferritin defined as <12 μg/L; ^g^ No CI available ^h^ Ferritin not measured in adult males. Abbreviations: NHANES—National Health and Nutrition Examination Survey (mean ferritin was assessed in the 2005–2006 cycle; prevalence of low ferritin was assessed in the 2003–2006 cycle); CI—confidence interval.

### 8.2. Zinc Status

While ferritin is an accepted measure of iron stores, there is no equivalent biochemical marker of zinc status. The most commonly used marker of zinc status is serum or plasma zinc [118]. Serum zinc reflects intakes of zinc but is maintained within a narrow range (12–15 µmol/L) and requires fasting blood samples for meaningful comparisons [119,120]. It is not considered a specific marker of zinc nutrition as circulating zinc is rapidly mobilized and turned over to meet tissue requirements [121]. Moreover, no reference ranges for serum or plasma zinc concentrations have been established for individuals, further limiting the diagnostic use of circulating zinc levels. The International Zinc Nutrition Consultative Group has suggested lower cut-offs of serum zinc for use in population studies based on the 2.5th percentile of NHANES II (1976–1980) participants [113], however given the difficulties with measuring a marker present in such minute quantities and the logistics of enforcing fasting status for blood sampling in national surveys, few countries measured zinc concentrations. As presented in Table 7, the US measured serum zinc in NHANES II (1976–1980) [121] and the UK measured plasma zinc in the 2008/09–2010/11 National Diet and Nutrition Survey [116]. Similar to serum ferritin, it appears males have higher zinc concentrations than females. Without an established biomarker, it is difficult to evaluate dietary zinc intakes in relation to biochemical status.

**Table 7:** Measurements of serum and plasma zinc in national surveys.

Country	Survey	Sample	Mean zinc (μmol/L)	2.5th Percentile (μmol/L)
US	NHANES II1976–1980 [121,122]	Males 10–64 years AM fasting (*n* = 1767)	15.0 ^a^	11.3
		Males 10–64 years AM non-fasting (*n* = 1750)	14.4 ^a^	10.7
		Males 10–64 years PM (*n* = 2765)	12.5 ^a^	9.3
		Females 10–70+ years AM fasting (*n* = 1576)	14.1 ^a^	10.7
		Females 10–70+ years AM non-fasting (*n* = 1658)	12.5 ^a^	10.1
		Females 10–70+ years PM (*n* = 2343)	11.6 ^a^	9.0
UK	2008/09–2010/11 UK National Diet and Nutrition Survey [116]	Males 19–64 years AM fasting (*n* = 160)	15.8 ^b^	10.3
		Females 19–64 years AM fasting (*n* = 231)	15.5 ^b^	10.0

^a^ Serum zinc; geometric mean; ^b^ Plasma zinc; arithmetic mean. Abbreviations: NHANES—National Health and Nutrition Examination Survey.

### 8.3. Correlation between Iron and Zinc Status

The value of using serum or plasma zinc as a measure of zinc status in small research studies is debatable [113], however a handful of studies have assessed correlations between iron and zinc status using such measures. Two studies have found positive correlations between serum or plasma zinc and serum ferritin [7,123], while two other studies have found no such association, but using categorical analysis, have found serum zinc concentrations to be lower among individuals with all-cause anemia [124,125]. Another study did not report assessment of correlation between plasma zinc and serum ferritin, but using categorical analysis found that serum ferritin was lower among participants with lower serum zinc [126]. Although evidence is limited, there appears to be a positive association between iron and zinc status, and as iron and zinc losses have different routes, this association may indicate a link between iron and zinc nutriture.

## 9. Effect of Iron Intake on Zinc Status and Zinc Absorption

It is often cited that serum zinc is decreased upon ingestion of over 25 mg elemental iron in a single dose, or when the molar ratio of iron content to zinc content in a meal or supplement is 2:1 or greater [3,127,128]. This notion is based on a series of studies that measured serum zinc hourly for four to six hours following ingestion of solutions or test foods (e.g., oysters) [127,129,130,131]. The applicability of these studies to balanced diets was later queried by Solomons [132] who pointed out that the molar ratio of iron to zinc in foods does not generally exceed 1:1, however these findings may have implications with the use of dietary supplements. Nevertheless, studies that have investigated the effect of long-term iron supplementation on serum zinc have yielded conflicting results. Some studies have found no effect of iron supplementation on serum zinc concentration (elemental iron dose ranging from 7.5 to 79 mg) [133,134,135,136,137], while others have found supplemental iron may impair serum zinc concentrations (elemental iron dose ranging from 18 to 60 mg) [138,139]. It should be noted that while some of these studies were conducted to assess zinc absorption, serum zinc data may not provide any meaningful information on zinc absorption but instead reflect redistribution of body zinc [140,141]. Absorption studies using isotope labeling have found that the iron to zinc molar ratio of meals has no bearing on the amount of zinc absorbed, and furthermore, that iron inhibits absorption of zinc when both are tested in solution, but this effect is diminished when food matrices are used [142,143,144]. Accordingly, it appears the iron concentration of foods does not affect zinc absorption, and the effect of ingesting supplementary iron on zinc status is unknown.

## 10. Effect of Zinc Intake on Iron Status and Iron Absorption

Studies have also investigated the reverse relationship of whether zinc intake can affect iron status, with some studies finding zinc supplementation (dose ranging from 10 to 25 mg/day elemental zinc taken from six weeks to six months) leads to a decrease in serum or plasma ferritin ranging from 18% to 69% [145,146,147,148], while others have found serum ferritin to increase by 45% to 51% [149,150]. However, a meta-analysis exploring an array of effects of zinc supplementation in children found zinc supplementation does not have an effect on serum or plasma ferritin, although the authors acknowledged significant heterogeneity among studies [151].

The few studies that have used isotope labeling to measure iron absorption in relation to zinc intake have yielded mixed results. Zinc has been found to inhibit absorption of iron when the micronutrients are ingested in solution [152,153,154], but the same effect has not been demonstrated in a food matrix [152,153]. The long-term effect of zinc supplementation on iron absorption is still unclear. Studies have observed no change in iron absorption from a solution before and after supplementation (two months of 20 mg/day elemental zinc) [155], as well as an increase in iron absorption from food of 97% following supplementation (six weeks of 22 mg/day elemental zinc) [145]. Similar to the effect of iron on zinc absorption, there is no evidence that consumption of zinc in foods affects iron absorption. Additionally, given the conflicting findings of existing research, the impact of supplementary zinc on iron status is yet to be determined.

## 11. Conclusions

The effects of iron intake on zinc status or zinc intake on iron status have not been consistently demonstrated. Meat, seafood, legumes and cereals tend to be sources of both dietary iron and dietary zinc. The contribution of cereals to intakes is noteworthy as cereal products are commonly fortified with iron and zinc. There is little regulation on such voluntary fortification practices, and few population nutrition surveys may be recent enough to reflect the current and unprecedented levels of fortification. Given the adverse consequences of excess iron and zinc consumption, assessment of the contribution of fortified foods to intakes is required. Across economically-developed nations, dietary recommendations for iron are higher in women and dietary recommendations for zinc are higher in men, reflecting the routes of loss. Across the same nations, women have tended to present with poorer iron and zinc status and are more likely to have inadequate iron stores. Despite claims that certain compounds inhibit and enhance absorption of both iron and zinc, there is insufficient evidence reflecting free-living populations to derive dietary recommendations, particularly in the case of zinc. Single-meal absorption studies have demonstrated that phytate, polyphenols in beverages, supplemental calcium and soy protein can inhibit iron absorption. Of these potential inhibitors, phytate and soy protein have been shown to also inhibit zinc absorption. Animal tissue and ascorbic acid have been found to enhance absorption of iron, however the effect of animal tissue on zinc absorption has not been convincingly tested and it appears ascorbic acid does not have an impact on zinc absorption. While single-meal studies have indicated that consumption of these compounds has a dramatic impact on iron absorption, these results are attenuated in longer-term absorption studies, and there is no consistent evidence that consumption of these compounds affects iron status. There is a lack of longer-term zinc absorption studies and studies assessing the impact of these compounds on zinc status.

Finally, it must be noted that discussion of national data was specific to English-speaking countries and these data may not be applied to other nations. Furthermore, animal models and *in vitro* studies have not been discussed, although these may provide further insight into iron and zinc nutriture. Despite the limitations of this review, topics for further research have been identified. Potential interactions need to be substantiated by studies investigating mixed diets in free-living samples, and these studies need to account for complex food matrices. Studies investigating whether iron from supplements inhibits zinc absorption or whether supplemental zinc inhibits iron absorption have been inconclusive, and no effects have been demonstrated in a food matrix. Further research is required to clarify the potential interactions between iron and zinc and other food components. Identification of a specific marker of zinc nutrition would advance understanding of dietary zinc, and research that integrates whole, varied diets with biomarker information is likely to indicate whether the dietary interactions involving iron and zinc have any influence on iron and zinc status. In the absence of such evidence, it may be prudent for individuals at risk of iron deficiency to maximize iron absorption by consuming potential absorption enhancers while avoiding inhibitors at mealtimes.
